# High resolution dermal ultrasound (US) combined with superficial radiation therapy (SRT) versus non-image guided SRT or external beam radiotherapy (XRT) in early-stage epithelial cancer: a comparison of studies

**DOI:** 10.1186/s12885-023-10577-z

**Published:** 2023-01-28

**Authors:** Lio Yu, Mairead Moloney, Songzhu Zheng, James Rogers

**Affiliations:** 1 Laserderm Dermatology, 327 Middle Country Rd, Smithtown, NY 11787 USA; 2 New York Institute of Technology College of Osteopathic Medicine, Old Westbury, NY USA; 3 Department of Applied Mathematics and Statistics, Stony Brook University, Stony Brook, NY USA; 4 Summit Analytical LLC, Denver, CO USA

**Keywords:** Non-melanoma skin cancer, Superficial radiation therapy, Image-guidance, Statistics, Local control, Comparison

## Abstract

**Background:**

To compare the effectiveness of high-resolution dermal ultrasound (US) guided superficial radiotherapy (SRT) to non-image-guided radiotherapy in the treatment of early-stage Non-Melanoma Skin Cancer (NMSC).

**Methods:**

A high-resolution dermal ultrasound (US) image guided form of superficial radiation therapy (designated here as US-SRT) was developed in 2013 where the tumor configuration and depth can be visualized prior to, during, and subsequent to treatments, using a 22 megahertz (MHz) dermal ultrasound (US) with a doppler component. We previously published the results using this technology to treat 2917 early-stage epithelial cancers showing a high local control (LC) rate of 99.3%. We compared these results with similar American studies from a comprehensive literature search used in an article/guideline published by American Society of Radiation Oncology (ASTRO) on curative radiation treatment of basal cell carcinoma (BCC), squamous cell carcinoma (SCC) and squamous cell carcinoma in-situ (SCCIS) lesions from 1988 to 2018. Only U.S. based studies with greater than 100 cases with similar patient/lesion characteristics and stages treated by external beam, electron, or superficial/orthovoltage radiation therapy were included in the criteria for selection. The resultant 4 studies had appropriate comparable cases identified and the data analyzed/calculated with regard to local control. Logistic regression analysis was performed comparing each study to US-SRT individually and collectively with stratification by histology (BCC, SCC, and SCCIS).

**Results:**

US-SRT LC was found to be statistically superior to each of the 4 non-image-guided radiation therapy studies individually and collectively (as well as stratified by histology subtype) with *p*-values ranging from *p* < 0.0001 to *p* = 0.0438.

**Conclusions:**

Results of US-SRT in local control were statistically significantly superior across the board versus non-image-guided radiation modalities in treatment of epithelial NMSC and should be considered a new gold standard for treatment of early-stage cutaneous BCC, SCC, and SCCIS.

## Background

Non-melanoma skin cancer (NMSC) is the most common cancer diagnosed in the United States and it is comprised mostly of basal cell carcinoma (BCC), squamous cell carcinoma (SCC), and squamous cell carcinoma in-situ (SCCIS) [1]. In 2012, NMSC incidence was estimated at 5·43 million NMSC lesions in 3·32 million individuals in the U.S [2]. It is estimated that the overall incidence of BCC increased by 145% between 1976–1984 and 2000–2010, and the overall incidence of SCC increased 263% over that same period [3].

Despite NMSC having a low mortality, high cure rates, rare metastasis, and only accounting for 0·1% of cancer deaths, the standard of treatment is surgical removal [1]. Surgical options consist of Mohs micrographic surgery (MMS), excision, shave removal, curettage, and electrodessication [4]. However, numerous nonsurgical, non-invasive modalities exist including topical treatments (i.e., imiquimod, 5-flurouracil [5-FU] etc.), cryotherapy, photodynamic therapy (PDT), laser therapy, and radiotherapy with several techniques available within each category. However, surgery, specifically MMS, has remained the mainstay of treatment as the literature promises the highest cure-rates at around 99% [5, 6].

Multiple radiation modalities exist for the treatment of NMSC, including brachytherapy, electron beam radiotherapy (EBRT), external radiation therapy (XRT), and superficial radiation therapy (SRT). Specifically, superficial radiation therapy, has been used by dermatologists for the past century to treat NMSC [7]. With the advent of MMS, this modality fell out of favor. However, more recently there has been advancements to SRT, including the use of a high frequency 22 megahertz (MHz) dermal ultrasound (US) to visualize superficial depth of the skin. This has led to the development of high-resolution dermal ultrasound image guided superficial radiotherapy, designated here as (US-SRT) and commercially known as image-guided SRT (IGSRT). Commercial units became available in 2014 that allowed for lesion visualization prior to, during, and after treatment. A recently published study by Yu et al. using US-SRT (“IGSRT”) to treat 2917 early-stage keratinocytic cancers yielded a high local control (LC) rate of 99·3% [8]. An updated abstract that added 93 patients and 133 lesions for a total of 1725 patients with 3050 early-stage keratinocytic lesions showed a continued high local control (LC) of 99·2% [9]. This suggests that US-SRT can offer comparable cure rates to that of the current “gold standard” treatment modality, namely MMS, for early-stage NMSC without needing surgery and its associated risks, discomfort, and cosmetic sequalae.^1^

### Objective

To statistically evaluate the local control (LC) differences, if any, of US-SRT versus non-image-guided radiotherapy modalities for the treatment of early-stage epithelial cancer.

## Methods

### Source information

US-SRT results have been investigated in two seminal studies referenced hereafter as the 2021 US-SRT study and the 2022 US-SRT study [8, 9]. Data from the 2021 US-SRT study was obtained via direct chart analysis of patients with histopathologic confirmed NMSC treated with US-SRT from multiple institutions. Data from the 2022 US-SRT study utilized the same data from the 2021 US-SRT study with the addition of 133 histopathological confirmed NMSC from 94 patients with updated follow-up intervals. Data on the additional 133 histopathologic confirmed NMSC in 94 patients was previously published [10].

US-SRT outcomes included in the present analyses are from a subset of the 2021 US-SRT study patients who have a follow-up of greater than 52 weeks; and are from the entire study population of the 2022 US-SRT study.

The American Society of Radiation Oncology (ASTRO) published a literature review containing 143 studies on curative radiation treatment for NMSC [11]. A subset of modern, pertinent, comparable studies that utilized superficial radiotherapy (SRT) and external radiotherapy (XRT) without image-guidance were identified as meeting the following inclusion/exclusion criteria.

#### Inclusion

Only studies performed in the USA.

#### Exclusion

Meta-analysis, brachytherapy, pre-operative, post-operative ± chemo/targeted agents used, recurrent or predominately recurrent, prior radiotherapy, T4 only/predominant, metastatic to parotids, involvement of parotids, wound healing only, no local control reported, perineural invasion in $\ge$ 50% of cases, and those lesions arising from scar.

These criteria resulted in four high-quality, recent, evidenced-based studies, each with greater than 100 subjects. The four studies provided the XRT/SRT outcome data for this study and are hereafter referenced as the Lovett study, Locke study, Silverman study, and Cognetta study [12–15].

### Local Control (LC) calculation

Local Control for this analysis was defined as complete resolution of the treated lesion without evidence of recurrence. Lesion counts for the two US-SRT studies and the four XRT/SRT studies were used to compute LC as “Number of lesions that did not recur / Total number of lesions”. Lesions were analyzed as independent events. Only T_is_, T_1_ and T_2_ lesions were included. Specifically, the 2 US-SRT studies only had T_is_, T_1_ and T_2_ lesions in the entire cohort whereas in the other studies, only early stage lesions of stage 0 to stage II( T_is_, T_1_ and T_2_) were included for analysis. This was done to allow proper comparisons (of “apples to apples”) so that better local control caused by higher proportion of favorable patients as a confounding factor is eliminated.

### Statistical analysis

Data availability in the two US-SRT studies and the four XRT/SRT studies made it possible to compare each US-SRT study (i.e., Yu study and Moloney study) to the following XRT/SRT studies: Lovett, Locke, Silverman, and Cognetta for BCC; Lovett, Locke, and Cognetta for SCC; and Cognetta for SCCIS. For each comparison, a logistic model was implemented that contained the effect of treatment with treatment levels defined as the studies under consideration. Odds ratios were then derived that compared the US-SRT studies to each available XRT/SRT study and to all XRT/SRT studies combined.

### Role of the funding source

The sponsor of the study (SkinCure Oncology) was not involved in the study design, collection, analysis, interpretation of data, or writing of the report. The decision to submit the paper for publication was solely that of Dr. Lio Yu and the co-authors.

## Results

Table 1 compares each of the US-SRT studies to each XRT/SRT study (Lovett, Locke, Silverman, and Cognetta).^2^ Comparisons using odds ratios from logistic regression models were made for BCC, SCC, and SCCIS, individually, with odds ratios greater than unity (1) favoring US-SRT. As indicated in Table 1, not all studies evaluated all histologic tumor categories (SCC, SCCIS, BCC), and therefore, only studies that included a given histologic tumor category could be used as a comparator study in the evaluation of that same histologic tumor category. Table 1 also reports comparisons by tumor category between each US-SRT study and all comparator XRT/SRT studies combined. Finally, the LC odds can be converted to a probability with an asymmetrical 95% confidence interval. For the 2021 and 2022 US-SRT studies, separately, LC probabilities and 95% confidence intervals were calculated for each US-SRT study. This allows an odds ratio comparison to be envisioned as a plotted difference in LC probabilities.

**Table 1:** Recurrence and Local Control Events for Basal Cell Carcinoma (BCC), Squamous Cell Carcinoma (SCC) and Squamous Cell Carcinoma In Situ (SCCIS) with High resolution dermal ultrasound image guided superficial radiotherapy (US-SRT) versus External Radiation Therapy (XRT) / Superficial Radiation Therapy (SRT) Contrasts Using Odds Ratios (OR)

Lesion	Treatment	Study	Outcome			Odds Ratio (XRT/SRT over US-SRT) (95% Confidence Limits)			
			**Recurrence**		**Local Control**				
			**(n)**	**(%)**	**(n)**	**Yu 2021: US-SRT**		**Moloney 2022: US-SRT**	
**BCC**	**US-SRT**	**Yu (2021)**	6	0.9	698	**OR**	***p*-value**	**OR**	***p*-value**
		**Moloney (2022)**	16	1.1	1471				
	**XRT/SRT**	**Lovett**	20	9.0	202	11.5 (4.6, 29.1)	< .0001	9.1 (4.6, 17.9)	< .0001
		**Locke**	21	6.4	305	8.0 (3.2, 20.0)	< .0001	6.3 (3.3, 12.3)	< .0001
		**Silverman**	52	6.0	810	7.5 (3.2, 17.5)	< .0001	5.9 (3.3, 10.4)	< .0001
		**Cognetta**	22	3.1	690	3.7 (1.5, 9.2)	0.0047	2.9 (1.5, 5.6)	0.0012
		**All**	115	5.4	2007	7.1 (3.1,16.3)	< .0001	5.6 (3.3, 9.6)	< .0001
**SCC**	**US-SRT**	**Yu (2021)**	4	0.7	544	**OR**	***p*-value**	**OR**	***p*-value**
		**Moloney (2022)**	7	0.8	926				
	**XRT/SRT**	**Lovett**	14	18.9	60	31.7 (10.1, 99.5)	< .0001	30.9 (12.0, 79.3)	< .0001
		**Locke**	15	15.2	84	24.3 (7.9, 74.9)	< .0001	23.6 (9.4, 59.5)	< .0001
		**Cognetta**	4	3.0	129	4.2 (1.0, 17.1)	0.0438	4.1 (1.2, 14.2)	0.0259
		**All**	33	10.8	273	14.8 (5.1, 43.3)	< .0001	14.4 (6.1, 33.9	< .0001
**SCCIS**	**US-SRT**	**Yu (2021)**	2	0.5	415	**OR**	***p*-value**	**OR**	***p*-value**
		**Moloney (2022)**	1	0.2	649				
	**XRT/SRT**	**Cognetta**	19	2.2	842	4.7 (1.1, 20.2)	0.0385	14.6 (2.0, 109.7)	0.0090

Silverman presented 2-year and 5-year follow-up results. Mean 5-year follow-up was used. Mean follow-up for Yu, Moloney, Lovett, Locke, and Cognetta, respectively, was 2·1, 2·1, 5, 5 and 2·6 years. Silverman presented no SCC data. Only Cognetta presented SCCIS

**Fig. 1:**
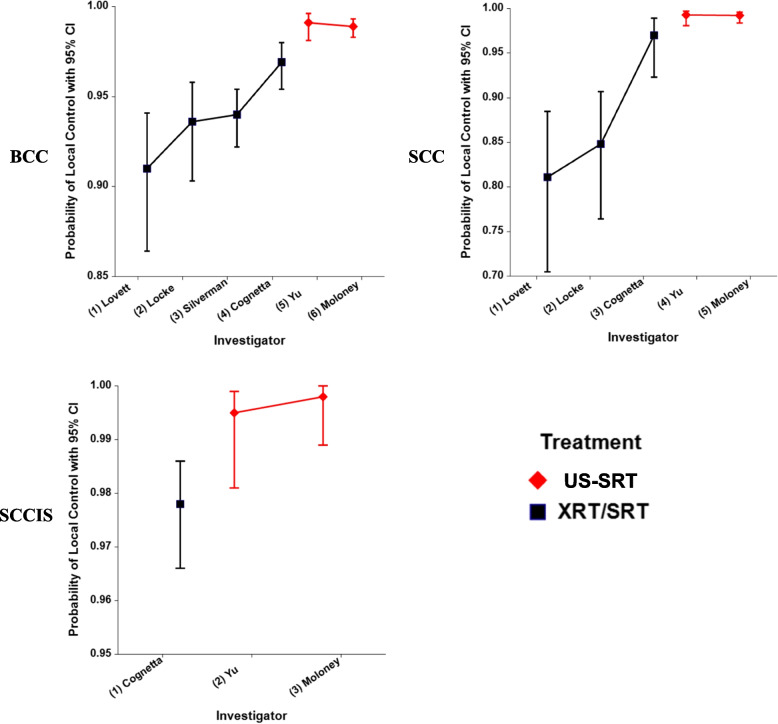
Basal Cell Carcinoma (BCC), Squamous Cell Carcinoma (SCC) and Squamous Cell Carcinoma In Situ (SCCIS) Probabilities of Local Control for the High resolution dermal ultrasound image guided superficial radiotherapy (US-SRT) and External Radiation Therapy (XRT) / Superficial Radiation Therapy (SRT) Investigations

Table 1 indicates that US-SRT LC was statistically superior to the comparator XRT/SRT studies individually, collectively, and stratified by histologic tumor type, with *p*-values ranging from *p* < 0·0001 to *p* = 0·0438. Figure 1 plots the LC probabilities for each US-SRT study compared to each XRT/SRT study separated by histology. Figure 1 affirms graphically the findings shown in Table 1.

## Discussion

Our analysis shows US-SRT confers a statistically significant improvement in local control for all histologic subtypes of epithelial NMSC (BCC, SCC, SCCIS) compared to all four high-quality, recent, large studies using non-image-guided forms of radiotherapy (XRT/SRT). The improvement in local control can be attributed to the image-guided component of superficial radiotherapy, as the high definition integrated dermal ultrasound with doppler features allows for visualization of the early-stage lesions’ depth, breadth, and overall configuration prior to, during, and after treatment. Visualization during treatment allows for the treatment provider to adjust radiotherapy dosages and energies of penetration daily if necessary. Visualization after treatment allows for confirmation of lesion resolution/response.

Advantages of US-SRT include high cure-rates as demonstrated in the 2021 and 2022 papers. It is cost effective, due to its low-recurrence rate. It offers cosmetic benefits as it is tissue sparing and the majority of NMSC lesions occur in cosmetically sensitive areas, such as the head and neck. Patients can avoid pain, scarring and risk of infection and/or bleeding since this is a non-surgical treatment modality. As many patients often have more than one lesion diagnosed simultaneously, multiple lesions can also be treated synchronously with US-SRT. Offices that use US-SRT have reported overall excellent (> 95%) patient satisfaction and provider satisfaction (internal data). Indeed, a wide variety of patients could benefit from US-SRT, especially those with lesions on the head and neck region such as the ear, nose, or eyelids for functional preservation purposes, on the face where cosmesis is of concern, as well as the scalp where surgical closure is challenging often requiring flap placement for larger or multiple adjacent/confluent lesions. In the below knee locations where poor vascularization can result in protracted non-healing, US-SRT has an advantage over surgery because of its non-invasive nature.

Absolute and relative contraindications to US-SRT, as previously described in the 2021 US-SRT studies, include lesion invasion to underlying bone or muscle, thickness > 6 mm, previous radiation to the same lesion site, ataxia telangiectasia, active connective tissue disease, active lupus or rheumatologic disease, concomitant management with radiation sensitizing chemotherapy agent, T4 stage, and node positive status [8].

US-SRT could be considered the preferred standard non-surgical radiotherapeutic treatment modality for appropriate patients with early-stage epithelial skin cancers (BCC, SCC, SCCIS) with comparable LC to MMS without the drawbacks of surgery. At the minimum, patients should be presented with the option to have their NMSC treated with US-SRT.

### Limitations

No randomized controlled trial exists for direct comparison of US-SRT to radiotherapy modalities, including superficial and external radiotherapy. The follow-up periods in this paper, though long enough to reasonably assure meaningful and accurate US-SRT to XRT/SRT comparisons, are unequal among studies.^3^

## Conclusion

Image guidance with high resolution dermal ultrasound in the form of US-SRT is shown to confer a statistically significant advantage in lesion local control over non- image guided forms of SRT or XRT in all subtypes of cutaneous epithelial NMSC and should be considered the preferred standard of non-surgical treatment for early stage cutaneous BCC, SCC, and SCCIS.

## Data Availability

Deidentified data are available on request from the corresponding author – Dr. Lio Yu at lio.yu@protonmail.com. Data will be available for request with publication for period of at least 1 year. Additional analyses may be available upon request from Dr. Lio Yu at lio.yu@protonmail.com, including but not limited to all odds and probabilities using logistic regression, conversion of some of the odds to probabilities with asymmetric confidence limits, raw percentages, and raw odds.

## Abbreviations

SRT: Superficial Radiotherapy/Superficial Radiation Therapy
MHz: Megahertz
LC: Local Control
ASTRO: American Society of Radiation Oncology
BCC: Basal Cell Carcinoma
SCC: Squamous Cell Carcinoma
SCCIS: Squamous Cell Carcinoma in-Situ
NMSC: Non-Melanoma Skin Cancer
MMS: Mohs Micrographic Surgery
PDT: Photodynamic Therapy
EBRT: Electron Beam Radiotherapy
XRT: External Radiation Therapy
IGSRT: Image-Guided Superficial Radiation Therapy

## Footnotes

1 ^A^ Per the American Academy of Dermatology practice guidelines, Mohs micrographic survey (MMS) local control is reported to have local control (LC) of 99% for basal cell carcinoma (BCC) and about 97% for cutaneous squamous cell carcinoma (SCC).^4,6^ The 5-year local recurrence rate for primary SCC lesions treated with MMS is reported to be 3.1%. The 5-year local recurrence rate for primary BCC lesions treated with MMS is reported to be 1%. Our reported high resolution dermal ultrasound image guided superficial radiotherapy LC is 99.1% (2021 US-SRT study^8^) and 98.9% (2022 US-SRT study^9^) for BCC and is 99.3% (2021 US-SRT study^8^) and 99.2% (2022 US-SRT study^9^) for SCC, and thus appears to be as good as MMS for BCC and potentially better for SCC.

2 ^B^ Minor differences may be present in the raw numbers of each histologic subtype [BCC, SCC, SCCIS] used in the original analysis by Yu et al. and the raw numbers used in this analysis.^8^ These differences can be attributed to variations in data filtering. For instance, follow-up data was calculated in days and converted to weeks and months, thus data may be filtered by follow-up time in days, weeks, or months. Additionally, seven lesions had multiple histologic subtypes and can be included in both histology categories in this analysis. Despite these minor differences in total lesion number per histologic category, the overall local control rate results remained substantively unchanged.

3 Both US-SRT studies (Yu et al*.* and Moloney et al*.*) had a mean follow-up of 2.1 years. The likelihood of local recurrence for early-stage NMSC is small after two years.^16,17^ Recurrence of BCC primarily occurs within the first 4–12 months.^18^ Thus, the variation in the average follow-up among the studies likely has little significance.
